# Ameliorative Effects of Raisin Polyphenol Extract on Oxidative Stress and Aging In Vitro and In Vivo via Regulation of Sirt1–Nrf2 Signaling Pathway

**DOI:** 10.3390/foods14010071

**Published:** 2024-12-30

**Authors:** Wenjing Gao, Caiyun Zhao, Xin Shang, Bin Li, Jintian Guo, Jingteng Wang, Bin Wu, Yinghua Fu

**Affiliations:** 1 College of Life Science and Technology, Xinjiang University, Urumqi 830017, China; gaowj0923@163.com (W.G.); zcy13565816792@163.com (C.Z.); sshangxin0919@163.com (X.S.); 18242180424@163.com (B.L.); guojintian99@163.com (J.G.); 18769520685@163.com (J.W.); 2 Institute of Agro-Products Storage and Processing, Xinjiang Academy of Agricultural Sciences, Urumqi 830091, China; xjuwubin0320@sina.com

**Keywords:** raisin polyphenols, oxidative stress, oxidatively damaged HepG2 cells, aging mice, Sirt1–Nrf2 signaling pathway

## Abstract

Raisins are an important source of polyphenolic compounds in plant foods, and polyphenols are associated with antioxidant and anti-aging activity. In this work, 628 polyphenols in raisin extracts were characterized using UPLC-MS/MS, mainly including tricetin 3′-glucuronide, diisobutyl phthalate, butyl isobutyl phthalate, isoquercitrin and 6-hydroxykaempferol-7-O-glucoside. The oxidative stress in H_2_O_2_-induced HepG2 cells and D-gal-induced aging mice was alleviated by raisin polyphenols (RPs) via increases in the cellular levels of superoxide dismutase (SOD), catalase (CAT) and glutathione (GSH), along with decreases in malonaldehyde (MDA), reactive oxygen species (ROS) and advanced glycosylation end-products (AGEs) levels. In addition, it was observed that RPs enhanced Sirt1 and Sirt3 expression, initiating the Keap1-Nrf2 signaling pathway, by upregulating the levels of nuclear Nrf2, facilitating the expressions of the antioxidant proteins NQO1 and HO-1, and downregulating Keap1 and cytoplasmic Nrf2 protein levels in H_2_O_2_-induced HepG2 cells and D-gal-induced aging mice. In summary, RP exerted antioxidant and anti-aging effects via regulating the Sirt1–Nrf2 signaling pathway in vitro and in vivo.

## 1. Introduction

Aging is generally categorized as the gradual deterioration of an organism over time, and the number and proportions of older people are continuously increasing [1,2]. According to the United Nations Population Division, the proportion of older persons (aged 60 or over) globally is projected to increase by 10.2% from 2010 to 2050, and the population aged 80 or over will increase by 2.5% [3]. The aging process is closely related to oxidative stress, which promotes cellular aging, leading to organismal senescence. Under normal physiological conditions, the organism’s oxidative and antioxidant systems are in a dynamic equilibrium relationship. The excessive production of ROS disrupts the organism’s redox system, causing oxidative stress and triggering chronic age-related diseases [4,5]. Therefore, the antioxidant and anti-aging activities of natural products have recently received increasing attention.

It has been shown that phenolic substances in plant foods have strong antioxidant effects [6,7]. It was detected that the sinapinic acid and salicylic acid among mustard seed polyphenols could scavenge free radicals and prevent H_2_O_2_-induced oxidative damage in HL-7702 cells [8]. Procyanidin B2 delayed D-gal-induced aging in mice through increasing the total antioxidant capacity (T-AOC), SOD and glutathione peroxidase (GSH-PX) activities and diminishing MDA levels in liver and brain tissues [9]. Raisins are dehydrated through solar heating or artificial heating and are full of nutrients, including polyphenols, sugars and a variety of vitamins and minerals [10,11]. The polyphenols in raisins could prevent age-related neurodegenerative diseases, inhibit oxidative damage in the body, and have excellent antioxidant capacity [12,13].

Research has shown that decreased Sirt1 and Sirt3 protein expression is involved in various age-related maladies [14,15]. It was demonstrated that the Sirt1 and Sirt3 proteins could activate the transcription factor Nrf2, promote the entry of the Nrf2 protein into the nucleus, and activate the Nrf2 signaling pathway. The activation of Sirt1 improves many senescence-related diseases, and a reduction in Sirt3 levels leads to the accumulation of ROS, ultimately leading to cellular senescence [16,17]. Activated Nrf2 separated from Keap1 and entered into the nucleus, facilitating the production of the downstream antioxidant proteins NAD(P)H quinone oxidoreductase 1 (NQO1) and heme oxygenase (HO-1), which enhanced antioxidant enzyme activities and decreased ROS level [18,19]. An earthworm water extract was demonstrated to protect against H_2_O_2_-induced oxidative damage in HEK293 cells by enhancing Sirt1 expression, promoting Nrf2 entry into the nucleus and increasing antioxidant enzyme activities [20]. Honokiol strengthened the antioxidant potential in mice by upregulating Sirt3 and nuclear Nrf2 protein, increasing SOD and GSH activity, and decreasing the level of ROS [21]. Moreover, resveratrol regulated the Keap1-Nrf2 signaling pathway by diminishing ROS production, enhancing the entry of Nrf2 into the nucleus, and improving NQO1 and HO-1 enzymes [22]. The ginsenoside Rh2 could increase the expression of the Sirt1 protein, reduce the production of ROS, ameliorate H_2_O_2_-induced oxidative damage in porcine oocytes, and increase antioxidant capacity [23].

In this study, the polyphenols in raisins were analyzed using UPLC-MS/MS, and their antioxidant and anti-aging properties were systematically demonstrated. Firstly, the ability of raisin polyphenols to ameliorate the oxidative damage induced by H_2_O_2_ in HepG2 cells and the aging induced by D-gal in mice were investigated by determining the SOD and CAT activities, as well as the GSH and MDA contents, in HepG2 cells and the brain, liver and serum of mice. The ROS levels were detected in HepG2 cells and in the brain and liver of mice. Furthermore, the impacts of raisin polyphenols on the Sirt1–Nrf2 signaling pathway in oxidatively damaged HepG2 cells as well as in aged mice were detected via determining the Keap1, Nrf2, NQO1 and HO-1 levels and the expression of the Sirt1 and Sirt3 proteins. This research illustrated that the RPs had antioxidant and anti-aging effects, which could be used as an edible antioxidant to improve the body’s endogenous antioxidant defense and delay the aging process.

## 2. Materials and Methods

### 2.1. Chemicals and Reagents

Seedless white raisins were purchased from Turpan in Xinjiang, China. Folin–Ciocalteau reagent and gallic acid were purchased from Yuanye Biotechnology Co., Ltd. (Shanghai, China), and D-gal was acquired from Sigma Chemical Co. (St. Louis, MO, USA). L-ascorbic acid (Vitamin C, Vc) and Vitamin E were purchased from Yuanye Biotechnology Co., Ltd. (Shanghai, China). MTT was purchased from Beijing Solarbio Technology Co., Ltd. (Beijing, China). Pancreatin and PBS were purchased from Hyclone (Logan, UT, USA).

### 2.2. Preparation of Raisin Polyphenols

The raisin powder (2.5000 g, accurately weighed) was extracted with a 70% ethanol solution with a 1:20 solid/liquid ratio at 50 °C for 2.6 h. The product was ultrasonicated for 20 min to obtain the crude raisin polyphenol extract. Then, this crude extract was purified using AB-8 macroporous resin wet-filled glass columns, and the sample was adsorbed by AB-8 macroporous resin for 24 h. The impurities were eluted with three-times the column volume of distilled water, and the polyphenolic substances were eluted with three-times the column volume of 80% ethanol; they were then concentrated using a rotary evaporator at 40 °C. Finally, the purified lyophilized powder of dried raisin polyphenols was obtained using a freeze dryer (ALPHA1-2LDplus) and stored at −20 °C. The polyphenol content was determined using the Folin–Ciocalteau colorimetric method, and the standard curve of gallic acid was Y = 0.0054X + 0.0057, R^2^ = 0.9994.

polyphenol content (mg/g) = (C × V × D)/M(1)

where C is the concentration of the polyphenols in the raisins calculated from the standard curve, mg/mL; V is the volume of sample, mL; D is the sample dilution; and M is the mass of sample, g.

### 2.3. Analysis of Raisin Polyphenols Compositions Using UPLC-MS/MS

The composition of the raisin polyphenols was analyzed using UPLC-MS/MS [24]. In total, 50 mg of the sample was mixed with 2000 μL of 70% methanol, and the mixture was then centrifuged at 12,000 rpm for 3 min at 4 °C. The obtained supernatant was purified via 0.22 μm membrane filtration and analyzed using UPLC-MS/MS. Mobility phases: solution A: purified water (0.1% formic acid); solution B: acetonitrile (0.1% formic acid). The mass spectrometry detection parameters for 30 polyphenols are provided in Table S1.

### 2.4. Cell Viability Assay

HepG2 cells were obtained from the Xinjiang Key Laboratory of Bioresources and Genetic Engineering. The HepG2 cells were cultured in DMEM medium, containing 10% FBS (*v/v*) and 1% penicillin, and streptomycin (*v/v*) at 37 °C with 5% CO_2_. The cell viability was detected using the MTT assay. Firstly, HepG2 cells in the logarithmic growth phase were evenly dispersed at 1 × 10^4^ cells/well in 96-well plates (Labselect, Beijing Labgic Technology Co., Ltd., Beijing, China) and incubated for 24 h. Then, the medium was replaced with the corresponding concentrations of raisin polyphenol solution and Vc for 24 h, followed by incubation with 1.5 mM H_2_O_2_ for 24 h. Finally, 10 µL of MTT (5 mg/mL) was added, the plate was cultured in an incubator for 4 h at 37 °C with 5% CO_2_, and the dark-blue formazan was dissolved with 150 µL of DMSO. Then, the absorbance at 490 nm was determined. The equation used is presented below:

Cell viability (%) = (OD_experimental group_ − OD_blank group_)/OD_blank group_ (2)

### 2.5. Animals Treatment and Experimental Protocols

This study was conducted by the Ethical Committee on Animal Research of Xinjiang University (Approval No. XJUAE-2024-009). Eight-week-old male ICR mice were purchased from Xinjiang Medical University Experimental Animal Center (Approval No. SCXK (Xin) 2023-0001).

**Figure 1:**
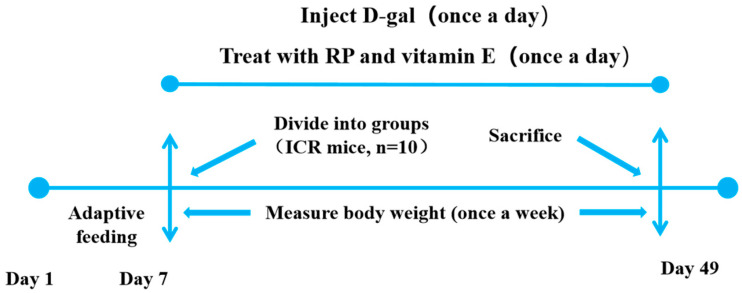
Animal experimental protocol.

Sixty mice were classified into six groups at random (n = 10): (1) normal control (NC) group, (2) D-gal model (MC) group, (3) vitamin E (VE) group, (4) low dosage of RPs (RP-L) group, (5) middle dosage of RPs (RP-M) group, and (6) high dosage of RPs (RP-H) group. The MC, VE and RP groups were intraperitoneally injected with 500 mg/(kg/bw) of D-gal, and the NC groups were administered an intraperitoneal injection of roughly saline equivalent (0.9%) once a day. Mice in the low-, middle- and high-dose RP groups received 100, 200 and 400 mg/(kg·bw) of RPs by oral gavage once a day. In the VE group, the mice were given 100 mg/(kg·bw) of VE once a day. The NC and MC groups were gavaged with 0.1 g/mL of saline (0.9%). Each week, the mice were weighed to adjust the injectable and oral-gavage dosages. The whole experiment lasted 42 days. The weights of the mice were recorded during the experiment. The whole experimental procedure is shown in Figure 1.

### 2.6. Preparation of Tissue and Serum Samples

After the final administration, all the animals were fasted for 24 h and sacrificed via cervical dislocation. The serum was collected by centrifuging the blood. Tissues were immediately retrieved and weighed. Essentially, 0.1000 g of animal tissues was homogenized with 0.9 mL of saline on ice. The homogenates were centrifuged at 3000 rpm for 10 min at 4 °C, and the supernatant was removed and stored for onward analyses. The formula for the organ index is provided below:

Organ index (mg/g) = organ weight (mg)/body weight (g)(3)

### 2.7. Hematoxylin and Eosin (HE) Staining

Brain and liver tissue was collected from the mice and fixed using a 4% paraformaldehyde solution. After paraffin embedding, sectioning and HE staining, the HE-stained sections of the brain tissues and livers of the mice in each group were observed.

### 2.8. Measurement of SOD, CAT, GSH, MDA, ROS and AGEs

The SOD, CAT, GSH, MDA, ROS and AGEs were detected, and the total protein levels were investigated using assay kits from Jiancheng Biotechnology Co., (Nanjing, China). The SOD activity was determined using the WST-1 method, indirectly determining the SOD activity by detecting the reaction of WST-1 with the superoxide anion. The CAT activity was determined using the ammonium molybdate method, in which H_2_O_2_ reacted with ammonium molybdate to form a light-yellow complex, and the absorbance value at 405 nm was measured to calculate the CAT activity. GSH reacts with dithiobinitrobenzoic acid (DTNB) to form a yellow compound, which was colorimetrically quantified as reduced glutathione (GSH) at 405 nm. The MDA content was determined using the TBA method. MDA could be condensed with thiobarbituric acid (TBA) to form a red product with a maximum absorption peak at 532 nm. The ROS level was detected using the DCFH-DA (2,7-dichlorofuorescin diacetate) probe. When ROS were present in the cell, DCFH was oxidized to DCF (dichlorofluorescein), a strong green, fluorescent substance. The AGEs levels were analyzed with an ELISA kit.

### 2.9. Measurement of the Expression of Sirt1, Sirt3, Keap1, Nrf2, NQO1 and HO-1

After treatment, proteins from the HepG2 cells were obtained with RIPA lysis buffer and then used for subsequent assays. The expressions of Sirt1, Sitr3, Keap1, Nrf2, NQO1 and HO-1 were analyzed with ELISA kits (Jiangsu Meimian Industrial Co., Ltd., Yancheng, China). The content of protein was measured with a BCA assay kit from Jiancheng Biotechnology Co., (Nanjing, China). The nuclear and cytoplasmic proteins were extracted using an assay kit from Beyotime Biotechnology Co., Ltd. (Shanghai, China).

### 2.10. Statistical Analysis

The data are represented as the means ± SDs. One-way ANOVA was performed using IBM SPSS Statistics 26, with *p* < 0.05 regarded as statistically significant, and GraphPad Prism 9.5 was used for graphing.

## 3. Results and Discussion

### 3.1. Analysis of Raisin Polyphenols (RP) via UPLC-MS/MS

**Figure 2:**
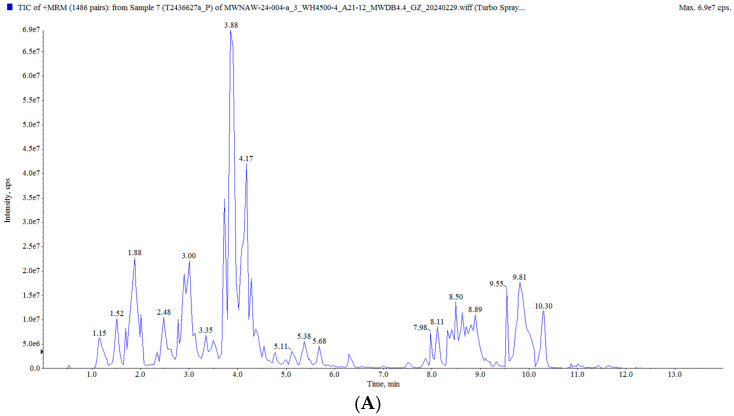

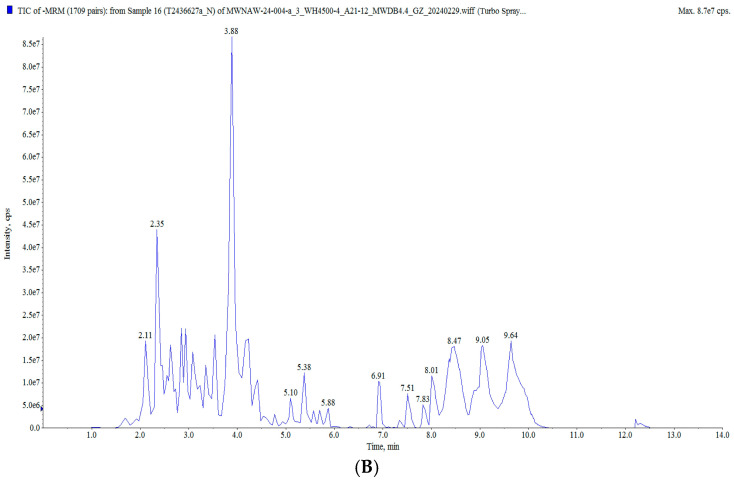
Total ion flow diagram of RP: (**A**) positive ion mode; (**B**) negative ion mode.

In this study, the raisin polyphenols (RPs) were purified using AB-8 macroporous resin, and the polyphenol content in the raisins was 117.82 ± 0.82 mg/g. The UPLC MS/MS analysis of the polyphenols in the raisins is presented in Figure 2 and Table S2. A total of 628 compounds were detected, classified into five groups: flavonoids, phenolic acids, stilbenes, lignans and coumarins, and tannins, mainly including tricetin 3′-glucuronide, diisobutyl phthalate, butyl isobutyl phthalate, isoquercitrin and 6-hydroxykaempferol-7-O-glucoside. Moreover, these polyphenol components have been characterized as bioactive substances with antioxidant and anti-aging activities. It was reported that isoquercitrin exerted a potential antioxidant effect by enhancing antioxidant enzyme activities and diminishing oxidative stress [25,26]. Quercetin-3-O-galactoside (isoquercitrin) improved the antioxidant capacity of an organism, reduced oxidative stress damage and prolonged the life span [27,28].

### 3.2. Effect of RPs on HepG2 Cell Cytotoxicity

**Figure 3:**
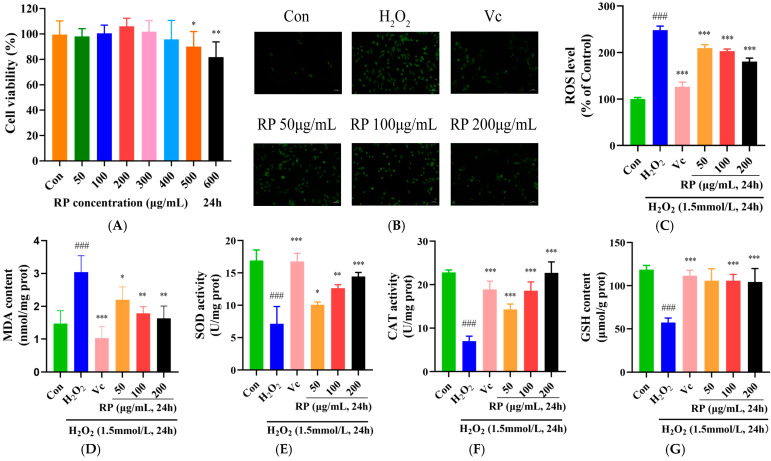
Effect of RPs on H_2_O_2_-induced HepG2 cells: (**A**) Cell viability; (**B**) HepG2 cells were stained with DCFH-DA. Scale bar: 50 μm; The fluorescence values were determined using a microplate reader for (**C**) ROS; (**D**) MDA; (**E**): SOD; (**F**) CAT; and (**G**) GSH. All the data are represented as the means ± SDs. *n* = 3 for each group. ### *p* < 0.001 vs. control group. * *p* < 0.05; ** *p* < 0.01; *** *p* < 0.001 vs. H_2_O_2_ group.

HepG2 cells were treated with RPs (50, 100, 200, 300, 400, 500 and 600 μmol/mL) for 24 h, and the cell viability was measured using the MTT method (Figure 3A). At concentrations of 50–400 μg/mL, the viability of HepG2 cells showed no significant change (*p* > 0.05), while it was significantly decreased at concentrations over 400 μg/mL (*p* < 0.05). Therefore, RPs at the range of 50–400 μg/mL exhibited no toxic effects on HepG2 cells, and 50, 100 and 200 μg/mL concentrations of RPs were selected for further experimentation.

### 3.3. RP’s Amelioration of Oxidative Damage Induced by H_2_O_2_ in HepG2 Cells

The accumulation of ROS causes cellular damage and disrupts the redox system, and antioxidant enzymes act as scavengers of ROS in the system to defend cells from oxidative stress [29,30]. As shown in Figure 3B–G, compared with those in the control group, ROS and MDA levels were significantly raised (*p* < 0.001), and the SOD, CAT activity and GSH content were significantly lessened (*p* < 0.001) in H_2_O_2_-induced HepG2 cells. After treatment with Vc and various doses of RP, the accumulation of ROS and MDA in oxidatively damaged HepG2 cells was significantly diminished (*p* < 0.05), and the levels of SOD and CAT were significantly elevated (*p* < 0.05). Meanwhile, Vc and RP (100 and 200 μg/mL) were able to significantly increase the GSH content (*p* < 0.001). It was demonstrated that Ilex paraguariensis extracts and its polyphenols could prevent oxidative damage and senescence in ARPE-19 cells by decreasing ROS levels and improving antioxidant enzyme activities [31]. The results suggested that treatment with Vc and different doses of RPs protected HepG2 cells from H_2_O_2_-induced oxidative damage via increasing the activity of antioxidant enzymes and decreasing the expression of MDA and ROS.

### 3.4. Effect of RPs on Sirt1–Nrf2 Signaling Pathway in H_2_O_2_-Induced HepG2 Cells

**Figure 4:**
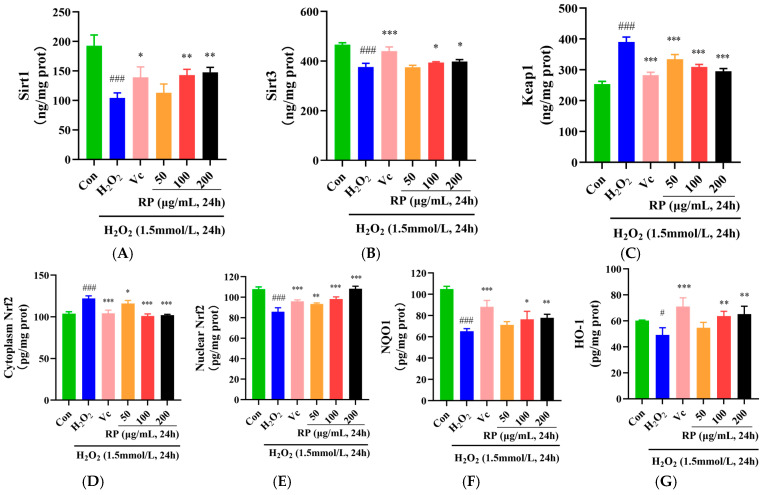
Effect of RPs on the Sirt1–Nrf2 signaling pathway in H_2_O_2_ -induced HepG2 cells: (**A**) Sirt1; (**B**) Sirt3; (**C**) Keap1; (**D**) cytoplasmic Nrf2; (**E**) nuclear Nrf2; (**F**) NQO1; (**G**) HO-1. All the data are represented as the means ± SDs. *n* = 3 for each group. # *p* < 0.05; ### *p* < 0.001 vs. control group. * *p* < 0.05; ** *p* < 0.01; *** *p* < 0.001 vs. H_2_O_2_ group.

In the Sirt1–Nrf2 signaling pathway, the dissociation of Keap1 and Nrf2 is stimulated by ROS, and the Nrf2 activated by Sirt1 and Sirt3 is released into the nucleus to regulate the antioxidant proteins NQO1 and HO-1 and increase the levels of SOD, CAT, and GSH [32,33]. As shown in Figure 4A,B, in contrast to the control group, the levels of the Sirt1 and Sirt3 proteins in oxidatively damaged HepG2 cells were significantly decreased (*p* < 0.001), and after treatment with Vc and RPs (100 μg/mL and 200 μg/mL), the Sirt1 and Sirt3 protein levels were significantly increased (*p* < 0.05). Moreover, the Keap1 and cytoplasmic Nrf2 protein levels were significantly elevated in the H_2_O_2_ group (*p* < 0.001) (Figure 4C), whereas the nuclear Nrf2 protein contents were significantly decreased (*p* < 0.001) (Figure 4D,E). After the administration of Vc and various doses of RP, the levels of the Keap1 and cytoplasmic Nrf2 proteins were significantly decreased (*p* < 0.05), while the nuclear Nrf2 protein contents were significantly increased (*p* < 0.01). Meanwhile, NQO1 and HO-1 were significantly diminished in oxidatively damaged HepG2 cells (*p* < 0.05) (Figure 4F,G), while the cellular NQO1 and HO-1 protein contents were significantly enhanced (*p* < 0.05) by Vc and RPs (100 and 200 μg/mL).

Furthermore, it was indicated that cocoa polyphenols could enhance the expression of Sirt1 and Sirt3, thereby alleviating H_2_O_2_-induced cellular senescence [34]. It was shown that polyphenols in argan oil activated the Nrf2 transcription factor, regulated the Nrf2 signaling pathway, and enhanced NQO1 and HO-1 levels, protecting cells from oxidative stress [35]. This work further confirmed that RPs could reduce Keap1 protein levels, upregulate the Nrf2 transcription factor, increase the NQO1 and HO-1 proteins, and activate the Sirt1–Nrf2 signaling pathway to enhance the cellular oxidation defense regime and protect cells against oxidative damage.

### 3.5. Effects of RPs on Body Weight and the Organ Index in D-Gal-Induced Aging Mice

**Figure 5:**
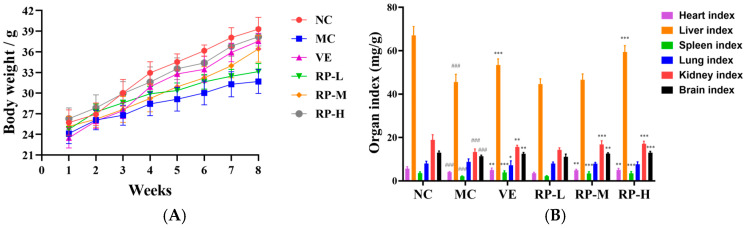
Effects of RPs on body weight (**A**) and organ index (**B**) in aging mice (*n* = 8 for each group). ### *p* < 0.001 vs. NC group. * *p* < 0.05; ** *p* < 0.01; *** *p* < 0.001 vs. MC group. NC: normal control group; MC: D-gal induced aging group; VE: vitamin E-treated group; RP-L: RP group (50 mg/kg); RP-M: RP group (100 mg/kg); RP-H: RP group (200 mg/kg).

As illustrated in Figure 5A, all the groups of mice showed an increase in body weight throughout the experimental period. Relative to the normal group, aging mice showed slow weight gain, and VE and RP administration reversed the reduction in body weight in the mice. Moreover, as shown in Figure 5B, the heart, liver, and brain tissue organ indexes were significantly increased in the VE, RP-M and RP-H groups (*p* < 0.01), and RP-H could significantly enhance the kidney indexes in aging mice (*p* < 0.05). Therefore, it was inferred that high concentrations of RPs might ameliorate organ atrophy in mice.

### 3.6. Histopathological Examination of Brain and Liver in Aging Mice

**Figure 6:**
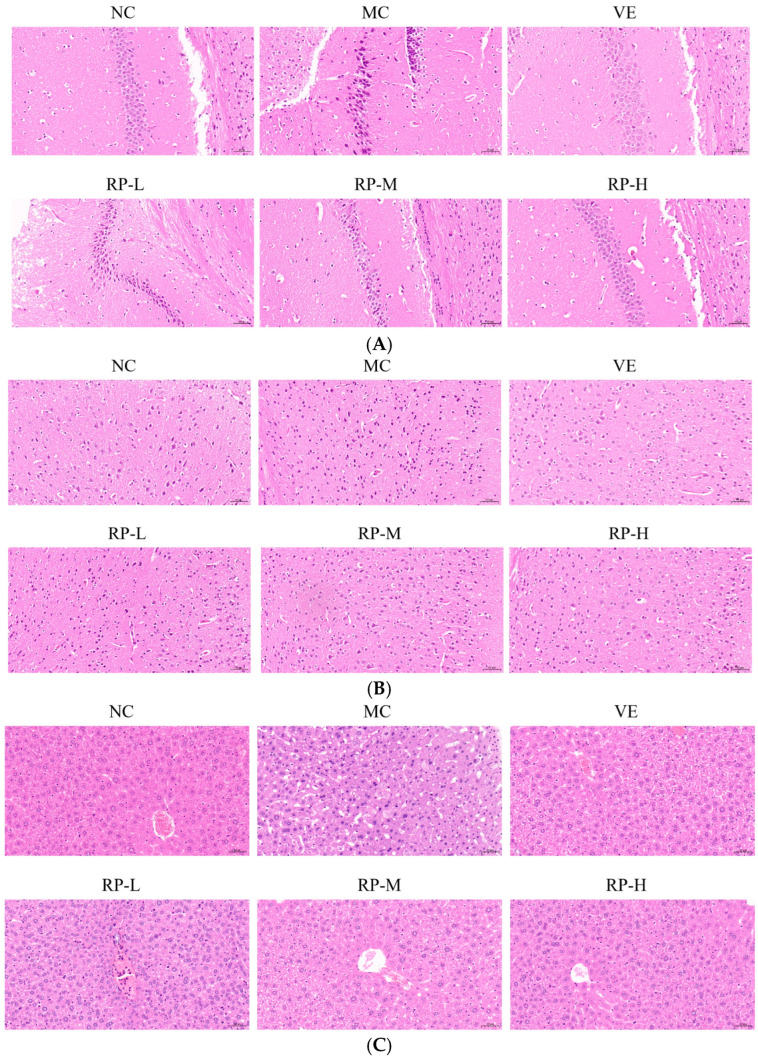
Effect of RPs on the histopathology in D-gal-induced aging mice: (**A**) hippocampus; (**B**) cerebral cortex; (**C**) liver. Scale bar: 50 µm. NC: normal control group; MC: D-gal induced aging group; VE: vitamin E-treated group; RP-L: RP group (50 mg/kg); RP-M: RP group (100 mg/kg); RP-H: RP group (200 mg/kg).

As illustrated in Figure 6A, the hippocampal cells of the mice in the NC group were tightly arranged, with clear staining and intact nuclei. The MC group mice showed a decreased number of nerve cells with a loosely arranged and irregular morphology compared with the NC group. After the VE and RP intervention, the hippocampal tissues of mice displayed varying amounts of ameliorative effects with a more orderly arrangement, and the nuclei were intact and neat. The results demonstrated that RP intervention preserves hippocampal neurons. Previous studies have shown that *Codonopsis pilosula* water extract could increase the number of neurons in the hippocampal region of D-gal-induced aging mice, resulting in a more organized arrangement and the protection of hippocampal neurons [36].

The morphology of the neurons of the cerebral cortex was normal in the NC group, with a uniform size (Figure 6B). In contrast, the cerebral cortex in the MC group exhibited neuronal loss and irregular morphology. The improvements in the VE, RP-M and RP-H groups contributed to attenuating neuronal loss, and there was an increase in the number of normal neurons in both groups. It was shown that RP treatment reversed D-gal-induced changes in the cerebral cortex to some extent. It has been shown that phenolics in fresh strawberries exhibited significant protective effects on the cerebral cortex, which showed numerous intact neurons and few cells showing neuronal degeneration and neuronophagia [37].

As shown in Figure 6C, the NC group of mice was observed to have a normal liver tissue structure, a normal nucleus morphology, clear boundaries and a neat arrangement, while the hepatocytes in the aging mice showed apparent pathological changes with enlarged gaps and altered nuclear morphology. The liver histomorphology showed different degrees of improvement upon the administration of VE and RP. This outcome illustrated that RPs mitigated D-gal-induced liver injury in aging mice. It was found that polyphenolic compounds in stevia residue could ameliorate liver cell degeneration and necrosis in aging mice, thereby alleviating liver damage induced by D-gal [38].

### 3.7. RPs Ameliorated Oxidative Stress in Aging Mice

**Figure 7:**
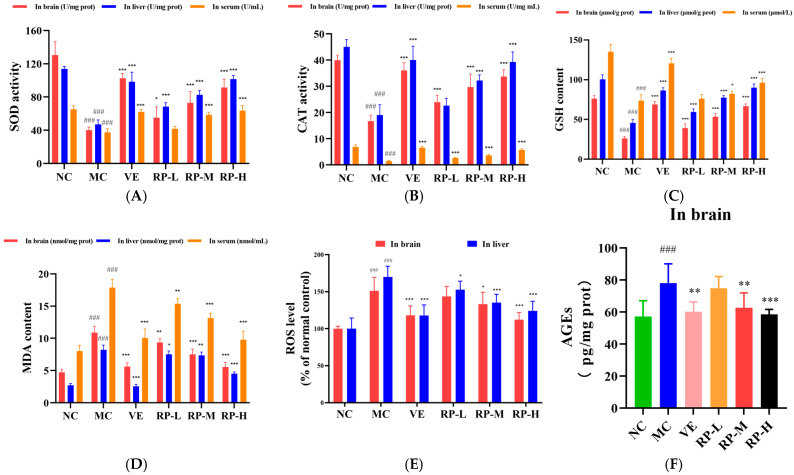
Effect of RPs on oxidative stress in aging mice: (**A**) SOD; (**B**) CAT; (**C**) GSH; (**D**) MDA; (**E**) ROS; (**F**) AGEs. All the data are represented as means ± SDs. *n* = 6 for each group. ### *p* < 0.001 vs. NC group. * *p* < 0.05; ** *p* < 0.01; *** *p* < 0.001 vs. MC group. NC: normal control group; MC: D-gal induced aging group; VE: vitamin E-treated group; RP-L: RP group (50 mg/kg); RP-M: RP group (100 mg/kg); RP-H: RP group (200 mg/kg).

As shown in Figure 7A–E, the levels of SOD, CAT, and GSH in the brains of the MC group of mice were significantly decreased (*p* < 0.001), and the ROS and MDA levels were significantly enhanced (*p* < 0.001). After the administration of VE and RP, the SOD, CAT, and GSH in the brain of aging mice were significantly improved (*p* < 0.05), and the MDA content was significantly reduced (*p* < 0.01). The RP-M and RP-H could significantly reduce the accumulation of ROS in the brain of aging mice (*p* < 0.05). Furthermore, the levels of SOD, CAT, and GSH in the liver of the MC group of mice were significantly lower (*p* < 0.001), and the ROS and MDA were significantly elevated (*p* < 0.001). After the administration of VE and various doses of RP, the SOD and GSH levels in aging mice were significantly enhanced (*p* < 0.001), and the contents of ROS and MDA were significantly decreased (*p* < 0.05). The RP-M and RP-H could significantly improve the CAT activity in the liver of aging mice (*p* < 0.001). Meanwhile, the levels of SOD, CAT, and GSH levels in the serum were also significantly diminished in aging mice (*p* < 0.001), while the MDA content was significantly increased (*p* < 0.001). After treatment with VE and various doses of RP, the activity of CAT in the serum of aging mice was significantly enhanced (*p* < 0.001), and the MDA level was significantly diminished (*p* < 0.01). The levels of SOD and GSH in the serum of aging mice were significantly enhanced in the RP-M and RP-H groups (*p* < 0.05). As shown in Figure 7F, compared to those in the NC group, the level of AGEs was significantly elevated in the brain of the MC group (*p* < 0.001), and compared to that in the MC group, the AGEs levels were significantly reduced in the VE, RP-M and RP-H groups (*p* < 0.01).

Previous studies showed that polyphenols in walnuts and chokeberries could improve the antioxidant enzymes of D-gal-induced senescent mice while reducing MDA levels in the liver [39]. Polyphenolic compounds in Litchi Pericarp could enhance the contents of SOD and GSH, reduce MDA levels in aging mice’s serum, and strengthen the antioxidant capacities of aging mice [40]. This indicated that the oxidative stress in D-gal-induced senescent mice was prevented by RPs, via increasing the levels of antioxidant enzymes to improve its antioxidant defense.

### 3.8. RPs Delayed Aging in Mice by Regulating the Sirt1–Nrf2 Signaling Pathway

**Figure 8:**
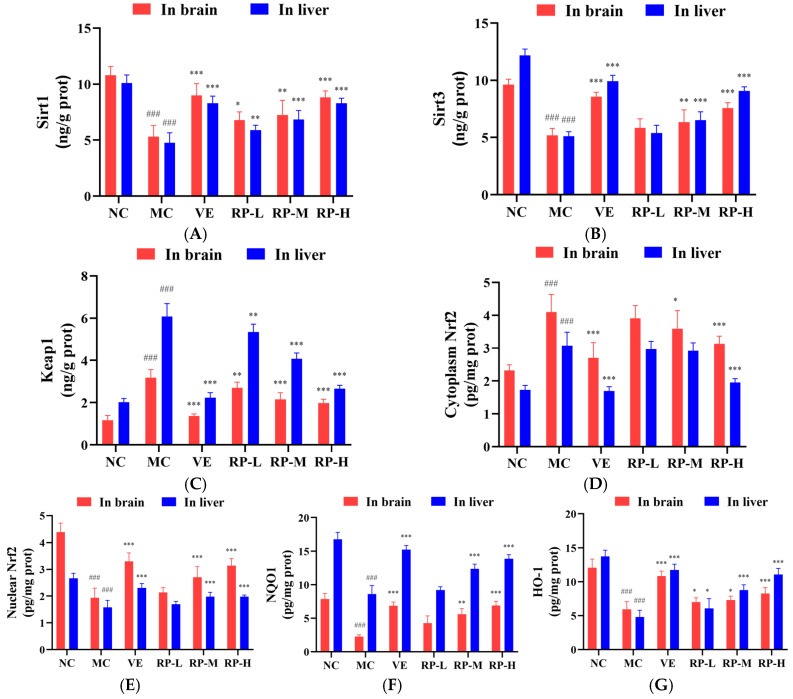
Effect of RPs on the Sirt1–Nrf2 signaling pathway in aging mice: (**A**) Sirt1; (**B**) Sirt3; (**C**) Keap1; (**D**) cytoplasmic Nrf2; (**E**) nuclear Nrf2; (**F**) NQO1; (**G**) HO-1. All the data are represented as the means ± SDs. *n* = 6 for each group. ### *p* < 0.001 vs. NC group. * *p* < 0.05; ** *p* < 0.01; *** *p* < 0.001 vs. MC group. NC: normal control group; MC: D-gal induced aging group; VE: vitamin E-treated group; RP-L: RP group (50 mg/kg); RP-M: RP group (100 mg/kg); RP-H: RP group (200 mg/kg).

As shown in Figure 8A–G, the levels of Sirt1, Sirt3, nuclear Nrf2, NQO1 and HO-1 proteins in the brain of the MC group of mice were significantly inhibited (*p* < 0.001), while the contents of Keap1 and cytoplasmic Nrf2 protein were significantly enhanced (*p* < 0.001). After the administration of VE and various doses of RP, the levels of Sirt1 and HO-1 were significantly elevated (*p* < 0.05), and the content of the Keap1 was significantly diminished (*p* < 0.01). The RP-M and RP-H could significantly enhance the Sirt3, nuclear Nrf2 and NQO1 contents (*p* < 0.01), while the cytoplasmic Nrf2 content was significantly reduced (*p* < 0.05). Meanwhile, compared to those in the NC group, the levels of Sirt1, Sirt3, nuclear Nrf2, NQO1 and HO-1 proteins in the liver of the MC group of mice were significantly reduced (*p* < 0.001), while the levels of Keap1 and cytoplasmic Nrf2 protein were significantly increased (*p* < 0.001). After the administration of VE and various doses of RPs, the levels of Sirt1 and HO-1 were significantly elevated (*p* < 0.05), and the content of Keap1 was significantly reduced (*p* < 0.01). The RP-M and RP-H could significantly improve the Sirt3, nuclear Nrf2 and NQO1 contents (*p* < 0.01). The cytoplasmic Nrf2 content was significantly reduced only in the RP-H group of mice (*p* < 0.001).

Recent studies indicated that the deacetylation of Nrf2 is facilitated by Sirt1, enhancing the stabilization of the Nrf2 transcription factor and thereby improving its antioxidant defenses [41,42]. It was found that the phenolic substances in *Scutellaria baicalensis* significantly regulated the protein expression levels of Keap1, Nrf2, HO-1 and NQO1 in the brains of aging mice [43]. It was shown that the flavonoids in *Garcinia mangostana* L. increased the expression of Sirt1 and promoted the entry of Nrf2 into the nucleus in the mouse liver, thus improving the antioxidant capacity of the mouse liver [44]. Our study demonstrated that RPs increased the Sirt1 and Sirt3 expressions and decreased the Keap1 protein level in senescent mice and enhanced the nuclear Nrf2 transcription factor level, activated the antioxidant proteins NQO1 and HO-1, and regulated the Sirt1–Nrf2 signaling pathway, thereby delaying the aging in mice.

## 4. Conclusions

In conclusion, this study showed that the polyphenolic compounds in raisins mainly included triecetin 3′-glucuronide, diisobutyl phthalate, butyl isobutyl phthalate, isoquercitrin and 6-hydroxykaempferol-7-O-glucoside. Raisin polyphenols (RPs) attenuated the oxidative stress in H_2_O_2_-induced HepG2 cells and D-gal-induced aging mice by reducing the levels of MDA, ROS accumulation and AGEs expression while also improving the activities of SOD, CAT and GSH content. Furthermore, the degrees of oxidative stress were reduced, and the process of aging was delayed by RPs in vitro and in vivo through regulating the Sirt1–Nrf2 signaling pathway. This was due to upregulating the Sirt1 and Sirt3 expressions, facilitating entry the Nrf2 transcription factor into the nucleus, and increasing the production of antioxidant proteins NQO1 and HO-1, along with downregulating the Keap1 and cytoplasmic Nrf2 contents. The results indicated that RPs had antioxidant and anti-aging effects and could be considered a natural product associated with the prevention of the aging process.

## Data Availability

The original contributions presented in this study are included in the article/Supplementary Materials. Further inquiries can be directed to the corresponding author.

## Supplementary Materials

The following supporting information can be downloaded at: https://www.mdpi.com/article/10.3390/foods14010071/s1, Table S1: Mass spectrometry detection parameters; Table S2: Main polyphenols in raisins.
