# Supplementary material for: Ameliorative Effects of Raisin Polyphenol Extract on Oxidative Stress and Aging In Vitro and In Vivo via Regulation of Sirt1–Nrf2 Signaling Pathway
Source: Foods. 2024 Dec 30;14(1):71. doi: 10.3390/foods14010071 (PMC11720718; doi:10.3390/foods14010071)
Supplement: Supplementary file 1 [file foods-14-00071-s001.zip › foods-3349225-supplementary.pdf]

Supplementary Material

Table S1. Mass spectrometry detection parameters

No.	Compounds	Formula	Retention time (min)	Declustering potential	Collision energy	Ionization model
1	Tricetin 3'-glucuronide	$C_{21}H_{18}O_{13}$	3.9	-50	-30	[M-H]-
2	Diisobutyl phthalate*	$C_{16}H_{22}O_4$	9.6	50	30	[M+H]+
3	Butyl isobutyl phthalate*	$C_{16}H_{22}O_4$	9.6	50	30	[M+H]+
4	Quercetin-3-O-glucoside (Isoquercitrin)*	$C_{21}H_{20}O_{12}$	4	50	30	[M+H]+
5	6-Hydroxykaempferol-7-O-glucoside	$C_{21}H_{20}O_{12}$	3.6	40	20	[M+H]+
6	Quercetin-3-O-alloside; Isohyperoside*	$C_{21}H_{20}O_{12}$	3.9	50	30	[M+H]+
7	Quercetin-3-O-galactoside (Hyperin)*	$C_{21}H_{20}O_{12}$	3.9	50	30	[M+H]+
8	Quercetin-4'-O-glucoside (Spiraeoside)*	$C_{21}H_{20}O_{12}$	3.8	-20	-30	[M-H]-
9	Phthalic anhydride	$C_8H_4O_3$	9.7	50	30	[M+H]+
10	Homoeriodictyol 7-O-glucoside*	$C_{22}H_{24}O_{11}$	4.2	50	30	[M+H]+
11	Quercetin-5-O-β-D-glucoside*	$C_{21}H_{20}O_{12}$	3.9	-50	-30	[M-H]-
12	6-Hydroxyluteolin 5-glucoside*	$C_{21}H_{20}O_{12}$	3.6	-50	-30	[M-H]-
13	Quercetin-7-O-glucoside*	$C_{21}H_{20}O_{12}$	4	-80	-30	[M-H]-
14	Hesperetin-5-O-glucoside	$C_{22}H_{24}O_{11}$	3.8	-60	-30	[M-H]-
15	Herbacetin-3-O-glucuronide*	$C_{21}H_{18}O_{13}$	3.6	50	30	[M+H]+
16	Delphinidin-3-O-glucuronide	$C_{21}H_{19}O_{13}^{+}$	3.6	50	30	[M]+

Continue Table S1. Mass spectrometry detection parameters

17	Anthranilic Acid	$C_7H_7NO_2$	4	20	30	[M+H]+
18	Demethyl coniferin	$C_{15}H_{20}O_8$	2.3	-50	-30	[M-H]-
19	1-O-p-Coumaroyl-β-D-glucose*	$C_{15}H_{18}O_8$	3	-20	-20	[M-H]-
20	6-Hydroxy-4',5,7-trimethoxyflavanone; Hamiltone A	$C_{18}H_{18}O_6$	3.3	-50	-30	[M-H]-
21	Rhamnetin-3-O-Glucoside*	$C_{22}H_{22}O_{12}$	4	50	30	[M+H]+
22	Quercetin-3-O-rutinoside (Rutin)*	$C_{27}H_{30}O_{16}$	3.8	50	30	[M+H]+
23	Isorhamnetin-3-O-Glucoside*	$C_{22}H_{22}O_{12}$	4.1	50	30	[M+H]+
24	Quercetin-3-O-(4''-O-glucosyl)rhamnoside	$C_{27}H_{30}O_{16}$	3.9	50	30	[M+H]+
25	Nepetin-7-O-alloside	$C_{22}H_{22}O_{12}$	4.2	50	30	[M+H]+
26	Caffeic acid	$C_9H_8O_4$	3.4	-20	-20	[M-H]-
27	1-O-Gentisoyl-β-D-glucoside*	$C_{13}H_{16}O_9$	1.9	-30	-20	[M-H]-
28	Isorhamnetin-7-O-glucoside (Brassicin)*	$C_{22}H_{22}O_{12}$	4.2	50	30	[M+H]+
29	Cimidahurinine*	$C_{14}H_{20}O_8$	2.3	-20	-20	[M-H]-
30	5-Glucosyloxy-2-Hydroxybenzoic acid methyl ester	$C_{14}H_{18}O_9$	3.3	-50	-30	[M-H]-

Table S2. Analysis of raisin polyphenols

No.	Compounds	CAS	Class	Proportion(%)
1	Tricetin 3'-glucuronide	-	Flavonoids	4.065±0.002
2	Diisobutyl phthalate	84-69-5	Phenolic acids	3.334±0.003
3	Butyl isobutyl phthalate	17851-53-5	Phenolic acids	3.331±0.325
4	Quercetin-3-O-glucoside (Isoquercitrin)	482-35-9	Flavonoids	2.207±0.253
5	6-Hydroxykaempferol-7-O-glucoside	-	Flavonoids	1.963±0.344
6	Quercetin-3-O-alloside; Isohyperoside	35589-21-0	Flavonoids	1.912±0.403
7	Quercetin-3-O-galactoside (Hyperin)	482-36-0	Flavonoids	1.874±0.282
8	Quercetin-4'-O-glucoside (Spiraeoside)	20229-56-5	Flavonoids	1.856±0.620
9	Phthalic anhydride	85-44-9	Phenolic acids	1.836±0.504
10	Homoeriodictyol 7-O-glucoside	-	Flavonoids	1.836±0.305
11	Quercetin-5-O-β-D-glucoside	-	Flavonoids	1.760±0.535
12	6-Hydroxyluteolin 5-glucoside	-	Flavonoids	1.719±0.648
13	Quercetin-7-O-glucoside	491-50-9	Flavonoids	1.716±0.494
14	Hesperetin-5-O-glucoside	69651-80-5	Flavanones	1.701±0.474
15	Herbacetin-3-O-glucuronide	-	Flavonols	1.645±0.635
16	Delphinidin-3-O-glucuronide	-	Anthocyanidins	1.488±0.546

Continue Table S2. Analysis of raisin polyphenols

17	Anthranilic Acid	118-92-3	Phenolic acids	1.327±0.147
18	Demethyl coniferin	-	Phenolic acids	1.217±0.226
19	1-O-p-Coumaroyl-β-D-glucose	7139-64-2	Phenolic acids	1.152±0.468
20	6-Hydroxy-4',5,7-trimethoxyflavanone; Hamiltone A	6626-61-5	Flavanones	1.132±0.521
21	Rhamnetin-3-O-Glucoside	27875-34-9	Flavonols	1.128±0.611
22	Quercetin-3-O-rutinoside (Rutin)	153-18-4	Flavonols	1.124±0.458
23	Isorhamnetin-3-O-Glucoside	5041-82-7	Flavonols	1.098±0.477
24	Quercetin-3-O-(4''-O-glucosyl)rhamnoside	59262-54-3	Flavonols	1.096±0.391
25	Nepetin-7-O-alloside	-	Flavones	1.096±0.412
26	Caffeic acid	331-39-5	Phenolic acids	1.073±0.444
27	1-O-Gentisoyl-β-D-glucoside	23445-11-6	Phenolic acids	1.071±0463
28	Isorhamnetin-7-O-glucoside (Brassicin)	6743-96-0	Flavonols	1.066±0.420
29	Cimidahurinine	142542-89-0	Phenolic acids	1.057±0.452
30	5-Glucosyloxy-2-Hydroxybenzoic acid methyl ester	-	Phenolic acids	1.005±0.663
